# Crystal structures of *N*-(4-chloro­phen­yl)-2-[(4,6-di­amino­pyrimidin-2-yl)sulfan­yl]acetamide and *N*-(3-chloro­phen­yl)-2-[(4,6-di­amino­pyrimidin-2-yl)sulfan­yl]acetamide

**DOI:** 10.1107/S2056989017003243

**Published:** 2017-03-03

**Authors:** S. Subasri, Timiri Ajay Kumar, Barij Nayan Sinha, Venkatesan Jayaprakash, Vijayan Viswanathan, Devadasan Velmurugan

**Affiliations:** a Centre of Advanced Study in Crystallography and Biophysics, University of Madras, Guindy Campus, Chennai 600 025, India; b Department of Pharmaceutical Sciences & Technology, Birla Institute of Technology, Mesra, Ranchi 835 215, Jharkhand, India

**Keywords:** crystal structure, di­amino­pyrimidin(2-yl) derivatives, thio­acte­amide, hydrogen bonding, C—H⋯π inter­actions, offset π–π inter­actions

## Abstract

The title compounds, C_12_H_12_ClN_5_OS, (I), and C_12_H_12_ClN_5_OS, (II), are 2-[(di­amino­pyrimidin-2-yl)sulfan­yl]acetamides. Compound (II), crystallizes with two independent mol­ecules (*A* and *B*) in the asymmetric unit. In each of the mol­ecules, in both (I) and (II), an intra­molecular N—H⋯N hydrogen bond forms an *S*(7) ring motif. The pyrimidine ring is inclined to the benzene ring by 42.25 (14)° in (I), and by 59.70 (16) and 62.18 (15)° in mol­ecules *A* and *B*, respectively, of compound (II). In the crystal of (I), mol­ecules are linked by pairs of N—H⋯N hydrogen bonds, forming inversion dimers with an *R*_2_^2^(8) ring motif. The dimers are linked *via* bifurcated N—H⋯O and C—H⋯O hydrogen bonds, forming corrugated layers parallel to the *ac* plane. In the crystal of (II), the *A* mol­ecules are linked through N—H⋯O and N—H⋯Cl hydrogen bonds, forming layers parallel to (100). The *B* mol­ecules are also linked by N—H⋯O and N—H⋯Cl hydrogen bonds, also forming layers parallel to (100). The parallel layers of *A* and *B* mol­ecules are linked *via* N—H⋯N hydrogen bonds, forming a three-dimensional structure.

## Abstract 2

The title compounds, (I) and (II), are 2-[(di­amino­pyrimidin-2-yl)sulfan­yl]acetamides. The mol­ecules have a folded conformation, with the pyrimidine ring being inclined to the benzene ring by 42.25 (14)° in (I), and by 59.70 (16) and 62.18 (15)° in the two independent mol­ecules of compound (II).

## Chemical context   

Di­amino­pyrimidine derivatives are reported to be therapeutic agents towards anti-cancer activity, selectively inhibiting c-Fms kinase of M-CSF-dependent myeloid leukemia cells (Xu *et al.*, 2010 ▸). They have also shown anti­bacterial activity (Kandeel *et al.*, 1994 ▸), are potential anti­microbial (Holla *et al.*, 2006 ▸) and anti-AIDS agents (Nogueras *et al.*, 1993 ▸) and anti­viral agents (Hocková *et al.*, 2003 ▸, 2004 ▸) and have shown promise as immunosuppressants (Blumenkopf *et al.*, 2002 ▸). In this connection, the title 2-[(4,6-di­amino­pyrimidin-2-yl)sulfan­yl] based analogues have been synthesized as anti­viral agents against NS2B/NS3 Dengue protease. We report herein on the syntheses and crystal structures of the title compounds, (I) and (II).

## Structural commentary   

**Figure 1:**
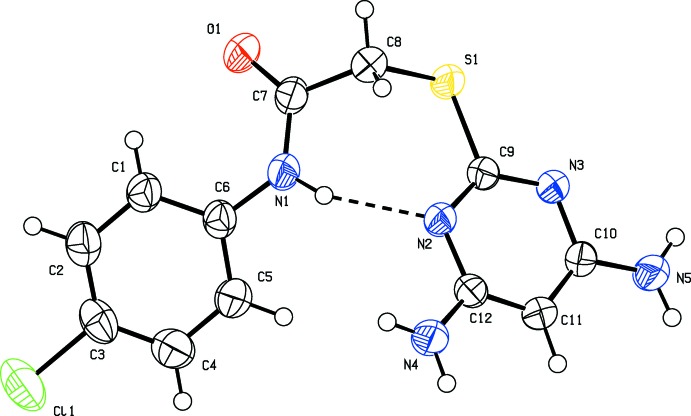
The mol­ecular structure of compound (I), with the atom labelling. Displacement ellipsoids are drawn at the 30% probability level. The intra­molecular N—H⋯N hydrogen bond is shown as a dashed lines (see Table 1 ▸).

The mol­ecular structures of compounds (I) and (II) are shown in Figs. 1 ▸ and 2 ▸, respectively. Compound (II) crystallizes with two independent mol­ecules (*A* and *B*) in the asymmetric unit, which have similar conformations (Fig. 3 ▸). The mol­ecules of both compounds are folded with the pyrimidine ring being inclined to the benzene ring by 42.25 (14)° in (I), and by 59.70 (16) and 62.18 (15)° in mol­ecules *A* and *B*, respectively, of compound (II). In compound (I), the N1—C7—C8—S1 torsion angle is 77.2 (3)°, while in mol­ecule *A* of compound (II), N5—C6—C5—S1 is 85.2 (3)°, and in mol­ecule *B* N10—C18—C17—S2 is 68.4 (3)°.
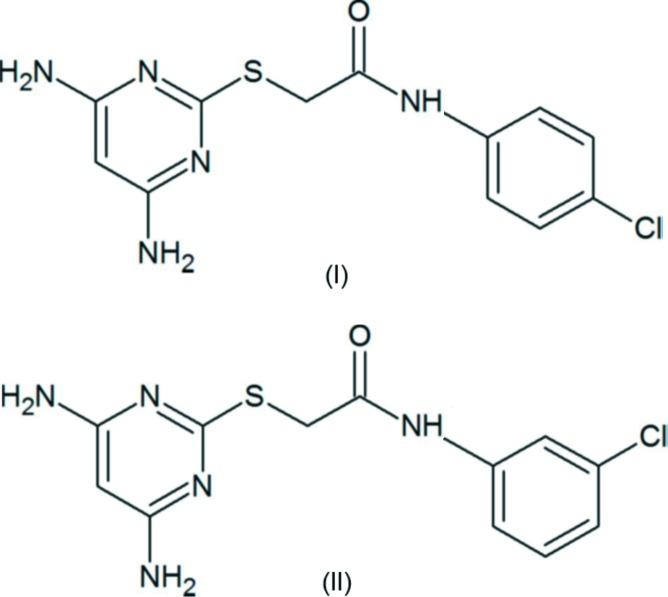


In compound (I), the intra­molecular N1—H1*A*⋯N2 hydrogen bond (Table 1 ▸) generates an *S*(7) ring motif, as shown in Fig. 1 ▸. Amine atoms N4 and N5 attached to the pyrimidine ring deviate by 0.018 (3) and 0.060 (3) Å, respectively. The chlorine atom Cl1 attached to the benzene ring deviates by 0.058 (1) Å.

**Figure 2:**
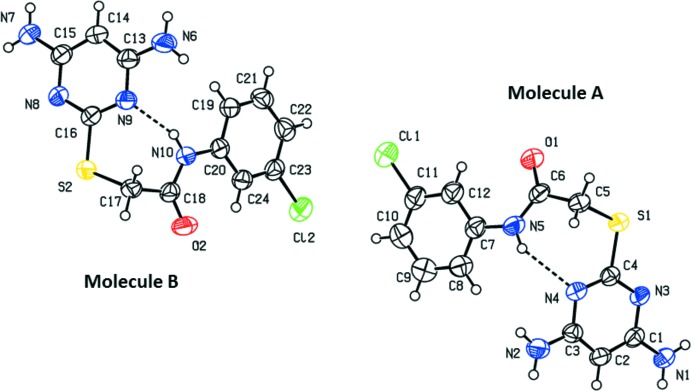
The mol­ecular structure of the two independent mol­ecules (*A* and *B*) of compound (II), with the atom labelling. Displacement ellipsoids are drawn at the 30% probability level. The intra­molecular N—H⋯N hydrogen bonds are shown as dashed lines (see Table 2 ▸).

**Figure 3:**
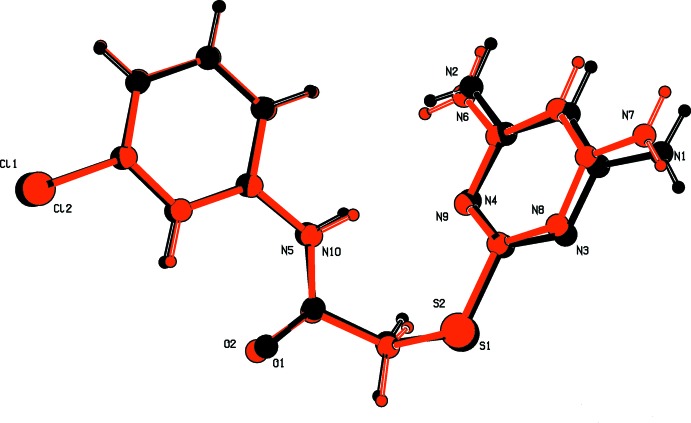
An AutoMolFit (*PLATON*; Spek, 2009 ▸) view of mol­ecule *B* (red) on mol­ecule *A* (back) of (II).

In compound (II), intra­molecular N5—H5⋯N4 and N10—H10⋯N9 hydrogen bonds (Table 2 ▸ and Fig. 2 ▸) in mol­ecules *A* and *B*, respectively, also generate *S*(7) ring motifs. In mol­ecule *A*, the amine group atoms N1 and N2 attached to the pyrim­idine ring deviate by 0.006 (3) and 0.004 (3) Å, respectively. The chlorine atom Cl1 attached to the benzene ring deviates by 0.013 (1) Å. In mol­ecule *B*, the amine group atoms N6 and N7 attached to the pyrimidine ring deviate by −0.003 (3) and 0.050 (3) Å, respectively. Atom Cl2 attached to the benzene ring deviates by 0.074 (1) Å.

## Supra­molecular features   

**Figure 4:**
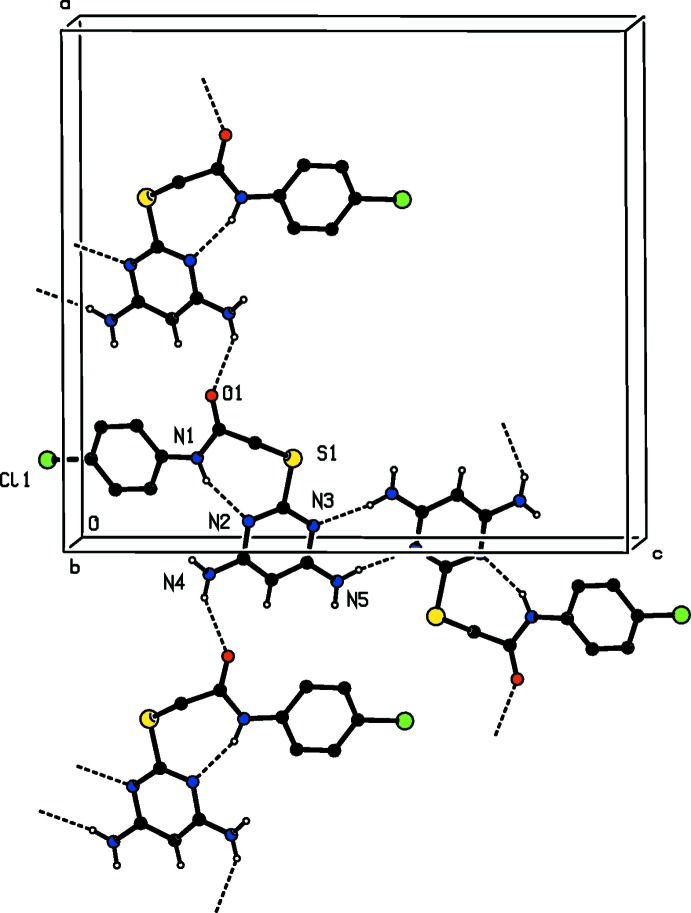
The crystal packing of compound (I) viewed along the *b* axis. H atoms not involved in hydrogen bonding (see Table 1 ▸), have been excluded for clarity.

**Figure 5:**
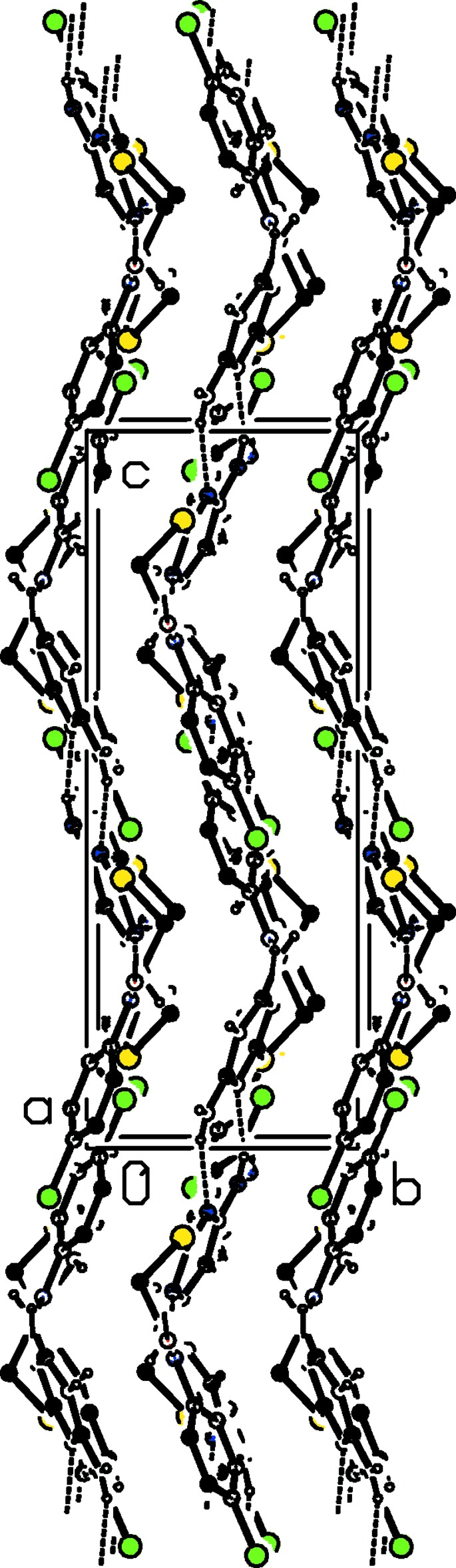
The crystal packing of compound (I) viewed along the *a* axis. H atoms not involved in hydrogen bonding (see Table 1 ▸), have been excluded for clarity.

In the crystal of (I), mol­ecules are linked by pairs of N—H⋯N hydrogen bonds, forming inversion dimers with an 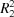
(8) ring motif (Table 1 ▸ and Fig. 4 ▸). The dimers are linked by *via* bifurcated N—H⋯O and C—H⋯O hydrogen bonds, forming corrugated layers parallel to the *ac* plane (Table 1 ▸ and Fig. 5 ▸).

**Figure 6:**
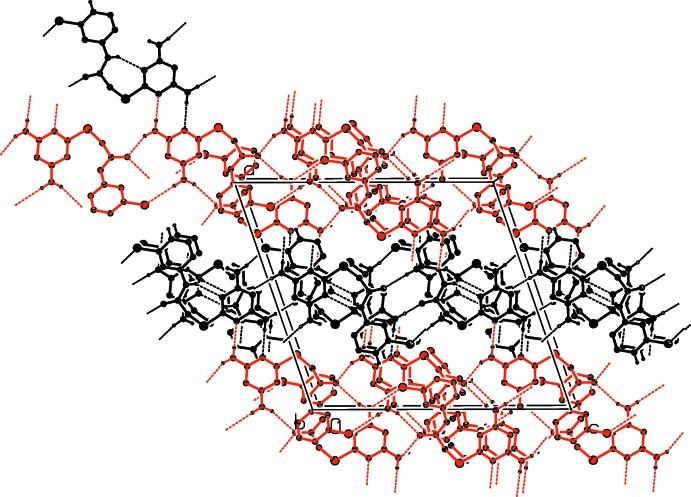
The crystal packing of compound (II) viewed along the *b* axis (colour code: *A* mol­ecules black, *B* mol­ecules red). H atoms not involved in hydrogen bonding (see Table 2 ▸), have been excluded for clarity.

In the crystal of (II), the *A* mol­ecules are linked through N—H⋯O and N—H⋯Cl hydrogen bonds, forming layers parallel to (100). Likewise the *B* mol­ecules are also linked by N—H⋯O and N—H⋯Cl hydrogen bonds, forming layers parallel to (100). The parallel layers of *A* and layers of *B* mol­ecules are linked *via* N—H⋯N hydrogen bonds, forming a three-dimensional structure (Table 2 ▸ and Fig. 6 ▸).

## Database survey   

A search of the Cambridge Structural Database (Version 5.37, update May 2016; Groom *et al.*, 2016 ▸) for 2-[(pyrimidine-2-yl)sulfan­yl]-*N*-phenyl­acetamide yielded five hits. Three of these involve (4,6-di­amino­pyrmidin-2-yl) groups. They include the 2-chloro­phenyl analogue, *N*-(2-chloro­phen­yl)-2-[(4,6-di­amino­pyrimidin-2-yl)sulfan­yl]acetamide (ARARUI; Subasri *et al.*, 2016 ▸). Here the pyrimidine and benzene rings are inclined to one another by 67.84 (6)°, compared to 42.25 (14)° in (I), and 59.70 (16) and 62.18 (15)° in mol­ecules *A* and *B*, respectively, of compound (II). As in the title compounds, there is also an intra­molecular N—H⋯N hydrogen bond present, stabilizing the folded conformation of the mol­ecule.

## Synthesis and crystallization   

**Compound (I):**

To a solution of 4,6-di­amino-pyrimidine-2-sulfanyl (0.5 g; 3.52 mmol) in 25 ml of ethanol, was added potassium hydroxide (0.2g; 3.52 mmol) and the mixture was refluxed for 30 min, after which 3.52 mmol of 2-chloro-*N*-(4-chloro­phen­yl)acetamide derivative was added and refluxed for 4 h. At the end of the reaction (monitored by TLC), the ethanol was evaporated *in vacuo* and cold water was added; the precipitate formed was filtered and dried to give compound (I) as a crystalline powder (yield 97%). Colourless block-like crystals were obtained from a solution in methanol and ethyl acetate (1:1) by slow evaporation of the solvents at room temperature.

**Compound (II):**

To a solution of 4,6-di­amino-pyrimidine-2-thiol (0.5 g; 3.52 mmol) in 25 ml of ethanol was added potassium hydroxide (0.2g; 3.52 mmol) and the mixture was refluxed for 30 min. Then 3.52 mmol of 2-chloro-*N*-(3-chloro­phen­yl)acetamide was added and refluxed for 3 h. At the end of the reaction (monitored by TLC), the ethanol was evaporated *in vacuo* and cold water was added and the precipitate formed was filtered and dried to give compound (II) as a crystalline powder (yield 92%). Colourless block-like crystals were obtained from a solution in methanol and ethyl acetate (2:1) by slow evaporation of the solvents at room temperature.

## Refinement   

Crystal data, data collection and structure refinement details are summarized in Table 3 ▸. For both (I) and (II), hydrogen atoms were placed in calculated positions and refined as riding: C—H = 0.93–0.97 Å and N—H = 0.86 Å, with *U*_iso_(H) = 1.2*U*_eq_(N,C).

## Supplementary Material

Crystal structure: contains datablock(s) global, I, II. DOI: 10.1107/S2056989017003243/su5348sup1.cif

Structure factors: contains datablock(s) I. DOI: 10.1107/S2056989017003243/su5348Isup4.hkl

Structure factors: contains datablock(s) II. DOI: 10.1107/S2056989017003243/su5348IIsup3.hkl

Supporting information file. DOI: 10.1107/S2056989017003243/su5348Isup4.cml

Supporting information file. DOI: 10.1107/S2056989017003243/su5348IIsup5.cml

CCDC references: 1534990, 1534989

Additional supporting information:  crystallographic information; 3D view; checkCIF report

## Figures and Tables

**Table 1:** Hydrogen-bond geometry (Å, °) for (I)

*D*—H⋯*A*	*D*—H	H⋯*A*	*D*⋯*A*	*D*—H⋯*A*
N1—H1*A*⋯N2	0.86	2.06	2.856 (3)	154
N5—H5*A*⋯N3^i^	0.86	2.16	2.990 (3)	162
N4—H4*B*⋯O1^ii^	0.86	2.22	2.969 (3)	146
C11—H11⋯O1^ii^	0.93	2.45	3.144 (3)	132

Symmetry codes: (i) 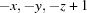; (ii) 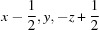.

**Table 2:** Hydrogen-bond geometry (Å, °) for (II)

*D*—H⋯*A*	*D*—H	H⋯*A*	*D*⋯*A*	*D*—H⋯*A*
N5—H5⋯N4	0.86	2.25	2.962 (3)	140
N10—H10*A*⋯N9	0.86	2.02	2.826 (3)	157
N1—H1*B*⋯O1^i^	0.86	2.19	2.931 (4)	145
N2—H2*B*⋯Cl1^i^	0.86	2.76	3.405 (3)	133
N6—H6*A*⋯O2^ii^	0.86	2.51	3.340 (4)	162
N6—H6*B*⋯Cl2^iii^	0.86	2.70	3.556 (3)	176
N7—H7*B*⋯O2^iii^	0.86	2.24	3.002 (4)	148
N1—H1*A*⋯N8^iv^	0.86	2.21	3.070 (4)	174
N7—H7*A*⋯N3^v^	0.86	2.19	3.046 (4)	178

Symmetry codes: (i) 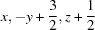; (ii) 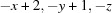; (iii) 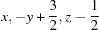; (iv) 
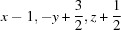
; (v) 
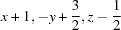
.

**Table 3:** Experimental details

	(I)	(II)
Crystal data		
Chemical formula	C_12_H_12_ClN_5_OS	C_12_H_12_ClN_5_OS
*M*_r_	309.78	309.78
Crystal system, space group	Orthorhombic, *P* *b* *c* *a*	Monoclinic, *P*2_1_/*c*
Temperature (K)	293	293
*a*, *b*, *c* (Å)	18.2743 (12), 7.4835 (5), 19.8021 (12)	18.220 (2), 8.1180 (12), 19.628 (2)
α, β, γ (°)	90, 90, 90	90, 108.761 (8), 90
*V* (Å^3^)	2708.1 (3)	2748.9 (6)
*Z*	8	8
Radiation type	Mo *K*α	Mo *K*α
μ (mm^−1^)	0.44	0.43
Crystal size (mm)	0.28 × 0.25 × 0.18	0.31 × 0.22 × 0.16

Data collection		
Diffractometer	Bruker SMART APEXII area-detector	Bruker SMART APEXII area-detector
Absorption correction	Multi-scan (*SADABS*; Bruker, 2008 ▸)	Multi-scan (*SADABS*; Bruker, 2008 ▸)
*T*_min_, *T*_max_	0.741, 0.863	0.742, 0.892
No. of measured, independent and observed [*I* > 2σ(*I*)] reflections	22685, 3372, 2007	25810, 6858, 3462
*R*_int_	0.060	0.075
(sin θ/λ)_max_ (Å^−1^)	0.669	0.668

Refinement		
*R*[*F* ^2^ > 2σ(*F* ^2^)], *wR*(*F* ^2^), *S*	0.050, 0.161, 1.08	0.050, 0.155, 0.96
No. of reflections	3372	6858
No. of parameters	181	361
H-atom treatment	H-atom parameters constrained	H-atom parameters constrained
Δρ_max_, Δρ_min_ (e Å^−3^)	0.42, −0.69	0.42, −0.42

Computer programs: *APEX2* and *SAINT* (Bruker, 2008 ▸), *SHELXS97* (Sheldrick, 2008 ▸), *SHELXL2016* (Sheldrick, 2015 ▸) and *PLATON* (Spek, 2009 ▸).

## supplementary crystallographic information

### (I) N-(4-Chlorophenyl)-2-[(4,6-diaminopyrimidin-2-yl)sulfanyl]acetamide Crystal data

**Table:**

C_12_H_12_ClN_5_OS	*D*_x_ = 1.520 Mg m^−^^3^
*M**_r_* = 309.78	Mo *K*α radiation, λ = 0.71073 Å
Orthorhombic, *P**b**c**a*	Cell parameters from 3372 reflections
*a* = 18.2743 (12) Å	θ = 2.1–28.4°
*b* = 7.4835 (5) Å	µ = 0.44 mm^−^^1^
*c* = 19.8021 (12) Å	*T* = 293 K
*V* = 2708.1 (3) Å^3^	Block, colourless
*Z* = 8	0.28 × 0.25 × 0.18 mm
*F*(000) = 1280	

### (I) N-(4-Chlorophenyl)-2-[(4,6-diaminopyrimidin-2-yl)sulfanyl]acetamide Data collection

**Table:**

Bruker SMART APEXII area-detector diffractometer	2007 reflections with *I* > 2σ(*I*)
Radiation source: fine-focus sealed tube	*R*_int_ = 0.060
ω and φ scans	θ_max_ = 28.4°, θ_min_ = 2.1°
Absorption correction: multi-scan (*SADABS*; Bruker, 2008)	*h* = −23→24
*T*_min_ = 0.741, *T*_max_ = 0.863	*k* = −9→9
22685 measured reflections	*l* = −26→26
3372 independent reflections	

### (I) N-(4-Chlorophenyl)-2-[(4,6-diaminopyrimidin-2-yl)sulfanyl]acetamide Refinement

**Table:**

Refinement on *F*^2^	Primary atom site location: structure-invariant direct methods
Least-squares matrix: full	Secondary atom site location: difference Fourier map
*R*[*F*^2^ > 2σ(*F*^2^)] = 0.050	Hydrogen site location: inferred from neighbouring sites
*wR*(*F*^2^) = 0.161	H-atom parameters constrained
*S* = 1.08	*w* = 1/[σ^2^(*F*_o_^2^) + (0.0795*P*)^2^ + 0.3384*P*] where *P* = (*F*_o_^2^ + 2*F*_c_^2^)/3
3372 reflections	(Δ/σ)_max_ < 0.001
181 parameters	Δρ_max_ = 0.42 e Å^−^^3^
0 restraints	Δρ_min_ = −0.69 e Å^−^^3^

### (I) N-(4-Chlorophenyl)-2-[(4,6-diaminopyrimidin-2-yl)sulfanyl]acetamide Special details

**Table:**

Geometry. All esds (except the esd in the dihedral angle between two l.s. planes) are estimated using the full covariance matrix. The cell esds are taken into account individually in the estimation of esds in distances, angles and torsion angles; correlations between esds in cell parameters are only used when they are defined by crystal symmetry. An approximate (isotropic) treatment of cell esds is used for estimating esds involving l.s. planes.

### (I) N-(4-Chlorophenyl)-2-[(4,6-diaminopyrimidin-2-yl)sulfanyl]acetamide Fractional atomic coordinates and isotropic or equivalent isotropic displacement parameters (Å^2^)

**Table:**

	*x*	*y*	*z*	*U*_iso_*/*U*_eq_	
C1	0.21682 (15)	0.1105 (4)	0.10059 (13)	0.0467 (7)	
H1	0.259655	0.165974	0.114873	0.056*	
C2	0.21206 (16)	0.0419 (4)	0.03543 (13)	0.0524 (7)	
H2	0.251506	0.052014	0.006002	0.063*	
C3	0.14875 (17)	−0.0409 (4)	0.01479 (13)	0.0523 (7)	
C4	0.08942 (17)	−0.0537 (4)	0.05714 (15)	0.0563 (8)	
H4	0.046632	−0.108550	0.042405	0.068*	
C5	0.09364 (15)	0.0152 (4)	0.12158 (14)	0.0505 (7)	
H5	0.053355	0.007491	0.150122	0.061*	
C6	0.15764 (13)	0.0963 (4)	0.14435 (12)	0.0411 (6)	
C7	0.21262 (13)	0.2114 (4)	0.25020 (12)	0.0417 (6)	
C8	0.18937 (14)	0.2955 (4)	0.31653 (13)	0.0466 (7)	
H8A	0.151615	0.383361	0.307401	0.056*	
H8B	0.230953	0.358263	0.335629	0.056*	
C9	0.06049 (13)	0.1256 (4)	0.36033 (12)	0.0370 (6)	
C10	−0.04825 (13)	0.0187 (4)	0.39846 (12)	0.0388 (6)	
C11	−0.07960 (13)	0.0629 (4)	0.33698 (12)	0.0423 (6)	
H11	−0.127989	0.033707	0.327503	0.051*	
C12	−0.03712 (13)	0.1511 (4)	0.29042 (12)	0.0384 (6)	
N1	0.15680 (11)	0.1680 (3)	0.20979 (10)	0.0439 (6)	
H1A	0.113996	0.186830	0.226380	0.053*	
N2	0.03568 (10)	0.1801 (3)	0.30081 (10)	0.0393 (5)	
N3	0.02360 (10)	0.0518 (3)	0.41104 (10)	0.0396 (5)	
N4	−0.06459 (12)	0.2133 (4)	0.23153 (10)	0.0513 (6)	
H4A	−0.036471	0.267317	0.203330	0.062*	
H4B	−0.110188	0.198752	0.222276	0.062*	
N5	−0.08599 (12)	−0.0543 (4)	0.44936 (11)	0.0533 (7)	
H5A	−0.064552	−0.077179	0.487041	0.064*	
H5B	−0.131697	−0.078337	0.444396	0.064*	
O1	0.27697 (10)	0.1893 (4)	0.23652 (10)	0.0662 (7)	
S1	0.15519 (3)	0.13935 (11)	0.37853 (3)	0.0458 (2)	
CL1	0.14411 (6)	−0.13289 (15)	−0.06572 (4)	0.0831 (4)	

### (I) N-(4-Chlorophenyl)-2-[(4,6-diaminopyrimidin-2-yl)sulfanyl]acetamide Atomic displacement parameters (Å^2^)

**Table:**

	*U*^11^	*U*^22^	*U*^33^	*U*^12^	*U*^13^	*U*^23^
C1	0.0401 (14)	0.061 (2)	0.0392 (13)	0.0034 (13)	0.0018 (11)	0.0079 (13)
C2	0.0559 (17)	0.060 (2)	0.0414 (14)	0.0041 (15)	0.0078 (13)	0.0057 (14)
C3	0.076 (2)	0.0448 (17)	0.0356 (13)	−0.0015 (15)	0.0010 (13)	0.0034 (12)
C4	0.0668 (19)	0.054 (2)	0.0478 (15)	−0.0159 (15)	−0.0061 (14)	0.0062 (15)
C5	0.0508 (16)	0.056 (2)	0.0446 (15)	−0.0121 (14)	0.0037 (12)	0.0070 (14)
C6	0.0404 (14)	0.0481 (17)	0.0349 (12)	0.0039 (12)	0.0025 (10)	0.0061 (12)
C7	0.0361 (13)	0.0500 (17)	0.0390 (13)	0.0010 (12)	0.0049 (11)	0.0045 (12)
C8	0.0396 (14)	0.0560 (18)	0.0441 (14)	−0.0070 (12)	0.0021 (11)	−0.0017 (13)
C9	0.0320 (12)	0.0451 (16)	0.0338 (11)	0.0015 (11)	0.0008 (9)	−0.0041 (11)
C10	0.0361 (13)	0.0442 (16)	0.0362 (12)	−0.0026 (11)	0.0054 (10)	−0.0040 (11)
C11	0.0329 (13)	0.0544 (18)	0.0396 (13)	−0.0040 (11)	−0.0016 (11)	−0.0011 (12)
C12	0.0347 (13)	0.0466 (17)	0.0339 (12)	0.0035 (11)	−0.0013 (10)	−0.0046 (11)
N1	0.0330 (11)	0.0617 (16)	0.0370 (11)	0.0029 (10)	0.0041 (9)	0.0006 (10)
N2	0.0307 (10)	0.0526 (14)	0.0344 (10)	0.0011 (9)	0.0008 (9)	0.0016 (10)
N3	0.0328 (11)	0.0517 (15)	0.0344 (10)	−0.0028 (9)	0.0001 (8)	0.0007 (10)
N4	0.0372 (12)	0.0783 (19)	0.0385 (11)	−0.0016 (12)	−0.0058 (10)	0.0095 (12)
N5	0.0426 (13)	0.0767 (19)	0.0405 (11)	−0.0161 (12)	−0.0008 (10)	0.0088 (12)
O1	0.0304 (10)	0.114 (2)	0.0538 (12)	0.0020 (11)	0.0062 (8)	−0.0101 (12)
S1	0.0312 (3)	0.0698 (5)	0.0363 (3)	−0.0015 (3)	−0.0016 (3)	0.0060 (3)
CL1	0.1216 (9)	0.0854 (7)	0.0422 (4)	−0.0171 (6)	0.0013 (4)	−0.0090 (4)

### (I) N-(4-Chlorophenyl)-2-[(4,6-diaminopyrimidin-2-yl)sulfanyl]acetamide Geometric parameters (Å, º)

**Table:**

C1—C6	1.390 (3)	C8—H8B	0.9700
C1—C2	1.391 (4)	C9—N2	1.327 (3)
C1—H1	0.9300	C9—N3	1.329 (3)
C2—C3	1.375 (4)	C9—S1	1.771 (2)
C2—H2	0.9300	C10—N5	1.338 (3)
C3—C4	1.374 (4)	C10—N3	1.359 (3)
C3—CL1	1.739 (3)	C10—C11	1.386 (3)
C4—C5	1.378 (4)	C11—C12	1.374 (4)
C4—H4	0.9300	C11—H11	0.9300
C5—C6	1.393 (4)	C12—N4	1.352 (3)
C5—H5	0.9300	C12—N2	1.364 (3)
C6—N1	1.403 (3)	N1—H1A	0.8600
C7—O1	1.218 (3)	N4—H4A	0.8600
C7—N1	1.337 (3)	N4—H4B	0.8600
C7—C8	1.517 (4)	N5—H5A	0.8600
C8—S1	1.807 (3)	N5—H5B	0.8600
C8—H8A	0.9700		
			
C6—C1—C2	120.1 (3)	H8A—C8—H8B	107.6
C6—C1—H1	120.0	N2—C9—N3	128.7 (2)
C2—C1—H1	120.0	N2—C9—S1	119.79 (18)
C3—C2—C1	119.7 (3)	N3—C9—S1	111.47 (17)
C3—C2—H2	120.2	N5—C10—N3	115.8 (2)
C1—C2—H2	120.2	N5—C10—C11	123.1 (2)
C4—C3—C2	120.9 (3)	N3—C10—C11	121.1 (2)
C4—C3—CL1	119.6 (2)	C12—C11—C10	118.1 (2)
C2—C3—CL1	119.5 (2)	C12—C11—H11	121.0
C3—C4—C5	119.7 (3)	C10—C11—H11	121.0
C3—C4—H4	120.2	N4—C12—N2	116.0 (2)
C5—C4—H4	120.2	N4—C12—C11	122.3 (2)
C4—C5—C6	120.7 (3)	N2—C12—C11	121.7 (2)
C4—C5—H5	119.7	C7—N1—C6	129.6 (2)
C6—C5—H5	119.7	C7—N1—H1A	115.2
C1—C6—C5	119.0 (2)	C6—N1—H1A	115.2
C1—C6—N1	123.7 (2)	C9—N2—C12	114.7 (2)
C5—C6—N1	117.2 (2)	C9—N3—C10	115.3 (2)
O1—C7—N1	124.8 (3)	C12—N4—H4A	120.0
O1—C7—C8	121.3 (2)	C12—N4—H4B	120.0
N1—C7—C8	113.9 (2)	H4A—N4—H4B	120.0
C7—C8—S1	114.6 (2)	C10—N5—H5A	120.0
C7—C8—H8A	108.6	C10—N5—H5B	120.0
S1—C8—H8A	108.6	H5A—N5—H5B	120.0
C7—C8—H8B	108.6	C9—S1—C8	103.73 (12)
S1—C8—H8B	108.6		
			
C6—C1—C2—C3	−0.4 (4)	O1—C7—N1—C6	−2.5 (5)
C1—C2—C3—C4	1.4 (5)	C8—C7—N1—C6	176.8 (3)
C1—C2—C3—CL1	−177.9 (2)	C1—C6—N1—C7	−22.2 (5)
C2—C3—C4—C5	−0.9 (5)	C5—C6—N1—C7	161.4 (3)
CL1—C3—C4—C5	178.4 (2)	N3—C9—N2—C12	−2.0 (4)
C3—C4—C5—C6	−0.5 (5)	S1—C9—N2—C12	175.36 (19)
C2—C1—C6—C5	−1.0 (4)	N4—C12—N2—C9	176.9 (2)
C2—C1—C6—N1	−177.3 (3)	C11—C12—N2—C9	−3.5 (4)
C4—C5—C6—C1	1.5 (4)	N2—C9—N3—C10	4.2 (4)
C4—C5—C6—N1	178.1 (3)	S1—C9—N3—C10	−173.28 (19)
O1—C7—C8—S1	−103.4 (3)	N5—C10—N3—C9	−179.5 (2)
N1—C7—C8—S1	77.2 (3)	C11—C10—N3—C9	−1.0 (4)
N5—C10—C11—C12	174.5 (3)	N2—C9—S1—C8	17.9 (2)
N3—C10—C11—C12	−3.8 (4)	N3—C9—S1—C8	−164.3 (2)
C10—C11—C12—N4	−174.2 (3)	C7—C8—S1—C9	−88.3 (2)
C10—C11—C12—N2	6.3 (4)		

### (I) N-(4-Chlorophenyl)-2-[(4,6-diaminopyrimidin-2-yl)sulfanyl]acetamide Hydrogen-bond geometry (Å, º)

**Table:**

*D*—H···*A*	*D*—H	H···*A*	*D*···*A*	*D*—H···*A*
N1—H1*A*···N2	0.86	2.06	2.856 (3)	154
N5—H5*A*···N3^i^	0.86	2.16	2.990 (3)	162
N4—H4*B*···O1^ii^	0.86	2.22	2.969 (3)	146
C11—H11···O1^ii^	0.93	2.45	3.144 (3)	132

Symmetry codes: (i) −*x*, −*y*, −*z*+1; (ii) *x*−1/2, *y*, −*z*+1/2.

### (II) N-(3-Chlorophenyl)-2-[(4,6-diaminopyrimidin-2-yl)sulfanyl]acetamide Crystal data

**Table:**

C_12_H_12_ClN_5_OS	*F*(000) = 1280
*M**_r_* = 309.78	*D*_x_ = 1.497 Mg m^−^^3^
Monoclinic, *P*2_1_/*c*	Mo *K*α radiation, λ = 0.71073 Å
*a* = 18.220 (2) Å	Cell parameters from 6858 reflections
*b* = 8.1180 (12) Å	θ = 1.2–28.4°
*c* = 19.628 (2) Å	µ = 0.43 mm^−^^1^
β = 108.761 (8)°	*T* = 293 K
*V* = 2748.9 (6) Å^3^	Block, colourless
*Z* = 8	0.31 × 0.22 × 0.16 mm

### (II) N-(3-Chlorophenyl)-2-[(4,6-diaminopyrimidin-2-yl)sulfanyl]acetamide Data collection

**Table:**

Bruker SMART APEXII area-detector diffractometer	3462 reflections with *I* > 2σ(*I*)
Radiation source: fine-focus sealed tube	*R*_int_ = 0.075
ω and φ scans	θ_max_ = 28.4°, θ_min_ = 1.2°
Absorption correction: multi-scan (*SADABS*; Bruker, 2008)	*h* = −24→24
*T*_min_ = 0.742, *T*_max_ = 0.892	*k* = −10→10
25810 measured reflections	*l* = −26→26
6858 independent reflections	

### (II) N-(3-Chlorophenyl)-2-[(4,6-diaminopyrimidin-2-yl)sulfanyl]acetamide Refinement

**Table:**

Refinement on *F*^2^	Primary atom site location: structure-invariant direct methods
Least-squares matrix: full	Secondary atom site location: difference Fourier map
*R*[*F*^2^ > 2σ(*F*^2^)] = 0.050	Hydrogen site location: inferred from neighbouring sites
*wR*(*F*^2^) = 0.155	H-atom parameters constrained
*S* = 0.96	*w* = 1/[σ^2^(*F*_o_^2^) + (0.0646*P*)^2^ + 0.6656*P*] where *P* = (*F*_o_^2^ + 2*F*_c_^2^)/3
6858 reflections	(Δ/σ)_max_ = 0.001
361 parameters	Δρ_max_ = 0.42 e Å^−^^3^
0 restraints	Δρ_min_ = −0.42 e Å^−^^3^

### (II) N-(3-Chlorophenyl)-2-[(4,6-diaminopyrimidin-2-yl)sulfanyl]acetamide Special details

**Table:**

Geometry. All esds (except the esd in the dihedral angle between two l.s. planes) are estimated using the full covariance matrix. The cell esds are taken into account individually in the estimation of esds in distances, angles and torsion angles; correlations between esds in cell parameters are only used when they are defined by crystal symmetry. An approximate (isotropic) treatment of cell esds is used for estimating esds involving l.s. planes.

### (II) N-(3-Chlorophenyl)-2-[(4,6-diaminopyrimidin-2-yl)sulfanyl]acetamide Fractional atomic coordinates and isotropic or equivalent isotropic displacement parameters (Å^2^)

**Table:**

	*x*	*y*	*z*	*U*_iso_*/*U*_eq_	
C1	0.43039 (17)	0.6518 (4)	0.39062 (15)	0.0438 (7)	
C2	0.50489 (17)	0.5880 (4)	0.41809 (15)	0.0461 (8)	
H2	0.533524	0.599968	0.466472	0.055*	
C3	0.53498 (16)	0.5069 (4)	0.37174 (15)	0.0424 (7)	
C4	0.42495 (16)	0.5606 (4)	0.27980 (15)	0.0400 (7)	
C5	0.42189 (17)	0.4300 (4)	0.14672 (16)	0.0485 (8)	
H5A	0.448033	0.345147	0.180668	0.058*	
H5B	0.385949	0.375940	0.105242	0.058*	
C6	0.48119 (18)	0.5233 (4)	0.12307 (16)	0.0473 (8)	
C7	0.61562 (17)	0.6320 (4)	0.17094 (16)	0.0476 (8)	
C8	0.67034 (19)	0.6788 (5)	0.23523 (18)	0.0592 (9)	
H8	0.663419	0.650619	0.278689	0.071*	
C9	0.7347 (2)	0.7666 (5)	0.2351 (2)	0.0737 (11)	
H9	0.770444	0.800365	0.278367	0.088*	
C10	0.7465 (2)	0.8045 (5)	0.1718 (2)	0.0663 (10)	
H10	0.790323	0.863055	0.171667	0.080*	
C11	0.69326 (19)	0.7553 (4)	0.10864 (18)	0.0554 (9)	
C12	0.62693 (19)	0.6684 (4)	0.10619 (18)	0.0538 (8)	
H12	0.591368	0.635782	0.062620	0.065*	
C13	1.07720 (18)	0.5427 (4)	−0.18594 (16)	0.0518 (8)	
C14	1.11114 (17)	0.6066 (4)	−0.23360 (16)	0.0545 (9)	
H14	1.085128	0.607744	−0.282867	0.065*	
C15	1.18621 (18)	0.6699 (4)	−0.20499 (16)	0.0504 (8)	
C16	1.18492 (16)	0.6063 (4)	−0.09333 (15)	0.0422 (7)	
C17	1.17491 (16)	0.5113 (4)	0.04034 (15)	0.0474 (8)	
H17A	1.152219	0.418732	0.009504	0.057*	
H17B	1.207528	0.467064	0.085940	0.057*	
C18	1.11047 (17)	0.6094 (4)	0.05351 (16)	0.0457 (7)	
C19	0.98833 (16)	0.7549 (4)	−0.01350 (15)	0.0428 (7)	
C20	0.93861 (17)	0.7880 (4)	−0.08218 (16)	0.0488 (8)	
H20	0.948465	0.742561	−0.121865	0.059*	
C21	0.87475 (18)	0.8876 (4)	−0.09216 (17)	0.0554 (9)	
H21	0.841809	0.907855	−0.138552	0.066*	
C22	0.85896 (18)	0.9577 (4)	−0.03444 (17)	0.0533 (8)	
H22	0.815921	1.024970	−0.041084	0.064*	
C23	0.90908 (17)	0.9246 (4)	0.03344 (16)	0.0477 (8)	
C24	0.97354 (17)	0.8249 (4)	0.04526 (16)	0.0460 (8)	
H24	1.006364	0.805075	0.091743	0.055*	
N1	0.39450 (15)	0.7294 (4)	0.43183 (13)	0.0584 (8)	
H1A	0.347948	0.765256	0.412896	0.070*	
H1B	0.418159	0.742920	0.477051	0.070*	
N2	0.60725 (15)	0.4414 (4)	0.39269 (13)	0.0573 (7)	
H2A	0.624904	0.396367	0.361454	0.069*	
H2B	0.635444	0.444816	0.437208	0.069*	
N3	0.38865 (13)	0.6359 (3)	0.31978 (12)	0.0436 (6)	
N4	0.49560 (13)	0.4945 (3)	0.30007 (12)	0.0412 (6)	
N5	0.55000 (14)	0.5449 (3)	0.17471 (13)	0.0501 (7)	
H5	0.554587	0.499513	0.215468	0.060*	
N6	1.00456 (16)	0.4803 (4)	−0.20701 (15)	0.0811 (11)	
H6A	0.984742	0.445292	−0.175429	0.097*	
H6B	0.978147	0.475742	−0.252009	0.097*	
N7	1.22370 (15)	0.7369 (4)	−0.24649 (14)	0.0698 (9)	
H7A	1.269792	0.775136	−0.227375	0.084*	
H7B	1.201739	0.741880	−0.292327	0.084*	
N8	1.22428 (13)	0.6664 (3)	−0.13364 (12)	0.0461 (6)	
N9	1.11350 (14)	0.5443 (3)	−0.11441 (12)	0.0479 (6)	
N10	1.05376 (13)	0.6562 (3)	−0.00640 (12)	0.0448 (6)	
H10A	1.058236	0.620508	−0.046127	0.054*	
O1	0.46540 (13)	0.5750 (4)	0.06196 (11)	0.0714 (7)	
O2	1.11150 (13)	0.6397 (3)	0.11485 (11)	0.0643 (7)	
S1	0.36824 (4)	0.56020 (11)	0.18798 (4)	0.0463 (2)	
S2	1.23535 (4)	0.62488 (11)	−0.00038 (4)	0.0492 (2)	
CL1	0.70803 (5)	0.80248 (14)	0.02776 (5)	0.0738 (3)	
CL2	0.89352 (5)	1.01784 (12)	0.10723 (5)	0.0649 (3)	

### (II) N-(3-Chlorophenyl)-2-[(4,6-diaminopyrimidin-2-yl)sulfanyl]acetamide Atomic displacement parameters (Å^2^)

**Table:**

	*U*^11^	*U*^22^	*U*^33^	*U*^12^	*U*^13^	*U*^23^
C1	0.0507 (17)	0.045 (2)	0.0406 (16)	−0.0074 (15)	0.0221 (14)	−0.0013 (14)
C2	0.0525 (17)	0.053 (2)	0.0330 (15)	−0.0094 (16)	0.0143 (13)	−0.0020 (15)
C3	0.0464 (16)	0.0413 (19)	0.0398 (16)	−0.0036 (14)	0.0143 (13)	0.0040 (14)
C4	0.0466 (16)	0.0371 (18)	0.0393 (15)	−0.0063 (14)	0.0178 (13)	−0.0023 (13)
C5	0.0539 (17)	0.052 (2)	0.0403 (16)	−0.0006 (16)	0.0166 (14)	−0.0116 (15)
C6	0.0552 (18)	0.055 (2)	0.0380 (16)	0.0100 (16)	0.0232 (15)	0.0024 (15)
C7	0.0521 (17)	0.046 (2)	0.0499 (18)	0.0132 (16)	0.0232 (15)	0.0032 (16)
C8	0.061 (2)	0.069 (3)	0.0482 (19)	0.0039 (19)	0.0180 (17)	0.0030 (18)
C9	0.066 (2)	0.089 (3)	0.062 (2)	−0.001 (2)	0.0159 (19)	−0.002 (2)
C10	0.054 (2)	0.073 (3)	0.069 (2)	−0.0075 (19)	0.0165 (19)	−0.003 (2)
C11	0.059 (2)	0.056 (2)	0.058 (2)	0.0111 (18)	0.0301 (17)	0.0080 (18)
C12	0.0582 (19)	0.055 (2)	0.0527 (19)	0.0064 (17)	0.0240 (16)	0.0013 (17)
C13	0.0544 (18)	0.059 (2)	0.0405 (17)	−0.0068 (17)	0.0129 (15)	−0.0037 (16)
C14	0.0539 (18)	0.075 (3)	0.0339 (16)	−0.0053 (18)	0.0128 (14)	−0.0001 (16)
C15	0.0532 (18)	0.059 (2)	0.0424 (17)	0.0088 (16)	0.0201 (15)	0.0042 (16)
C16	0.0478 (16)	0.0424 (19)	0.0394 (15)	0.0039 (15)	0.0182 (13)	0.0018 (14)
C17	0.0470 (16)	0.055 (2)	0.0384 (16)	−0.0037 (15)	0.0117 (13)	0.0106 (15)
C18	0.0487 (17)	0.050 (2)	0.0405 (17)	−0.0135 (15)	0.0176 (14)	−0.0007 (15)
C19	0.0411 (15)	0.0448 (19)	0.0436 (17)	−0.0150 (14)	0.0151 (13)	−0.0049 (15)
C20	0.0448 (17)	0.060 (2)	0.0409 (17)	−0.0111 (16)	0.0123 (14)	0.0017 (16)
C21	0.0499 (18)	0.064 (2)	0.0470 (18)	−0.0147 (17)	0.0079 (15)	0.0024 (17)
C22	0.0452 (17)	0.053 (2)	0.060 (2)	−0.0072 (16)	0.0147 (16)	0.0037 (17)
C23	0.0510 (17)	0.050 (2)	0.0471 (18)	−0.0123 (16)	0.0227 (15)	−0.0012 (15)
C24	0.0470 (17)	0.050 (2)	0.0417 (17)	−0.0109 (15)	0.0149 (14)	0.0024 (15)
N1	0.0530 (15)	0.086 (2)	0.0404 (14)	0.0043 (15)	0.0203 (12)	−0.0148 (14)
N2	0.0543 (15)	0.071 (2)	0.0427 (15)	0.0115 (15)	0.0108 (12)	0.0000 (14)
N3	0.0461 (13)	0.0513 (17)	0.0367 (13)	−0.0030 (12)	0.0180 (11)	−0.0083 (12)
N4	0.0487 (14)	0.0416 (15)	0.0345 (13)	0.0011 (12)	0.0153 (11)	−0.0003 (11)
N5	0.0552 (15)	0.0582 (19)	0.0387 (14)	0.0041 (14)	0.0178 (12)	0.0082 (13)
N6	0.0650 (18)	0.131 (3)	0.0406 (16)	−0.044 (2)	0.0086 (14)	−0.0034 (18)
N7	0.0558 (16)	0.114 (3)	0.0421 (15)	−0.0083 (17)	0.0198 (13)	0.0182 (16)
N8	0.0448 (13)	0.0557 (18)	0.0399 (14)	0.0009 (12)	0.0167 (11)	0.0077 (12)
N9	0.0468 (14)	0.0583 (18)	0.0372 (14)	−0.0109 (13)	0.0115 (11)	−0.0003 (13)
N10	0.0414 (13)	0.0570 (18)	0.0359 (13)	−0.0045 (12)	0.0126 (11)	−0.0002 (12)
O1	0.0594 (14)	0.113 (2)	0.0437 (13)	0.0075 (14)	0.0188 (11)	0.0222 (14)
O2	0.0714 (15)	0.0839 (18)	0.0362 (12)	0.0075 (14)	0.0152 (11)	−0.0032 (12)
S1	0.0464 (4)	0.0585 (6)	0.0351 (4)	0.0021 (4)	0.0146 (3)	−0.0024 (4)
S2	0.0415 (4)	0.0653 (6)	0.0391 (4)	−0.0092 (4)	0.0107 (3)	0.0047 (4)
CL1	0.0698 (6)	0.0948 (8)	0.0647 (6)	−0.0090 (5)	0.0327 (5)	0.0072 (5)
CL2	0.0665 (5)	0.0756 (7)	0.0602 (5)	0.0000 (5)	0.0308 (4)	−0.0049 (5)

### (II) N-(3-Chlorophenyl)-2-[(4,6-diaminopyrimidin-2-yl)sulfanyl]acetamide Geometric parameters (Å, º)

**Table:**

C1—N1	1.348 (4)	C15—N7	1.336 (4)
C1—N3	1.360 (3)	C15—N8	1.348 (4)
C1—C2	1.389 (4)	C16—N8	1.320 (3)
C2—C3	1.372 (4)	C16—N9	1.331 (4)
C2—H2	0.9300	C16—S2	1.766 (3)
C3—N2	1.355 (4)	C17—C18	1.508 (4)
C3—N4	1.362 (3)	C17—S2	1.808 (3)
C4—N3	1.327 (3)	C17—H17A	0.9700
C4—N4	1.332 (4)	C17—H17B	0.9700
C4—S1	1.766 (3)	C18—O2	1.223 (3)
C5—C6	1.510 (4)	C18—N10	1.346 (4)
C5—S1	1.800 (3)	C19—C24	1.388 (4)
C5—H5A	0.9700	C19—C20	1.387 (4)
C5—H5B	0.9700	C19—N10	1.405 (4)
C6—O1	1.215 (3)	C20—C21	1.378 (4)
C6—N5	1.346 (4)	C20—H20	0.9300
C7—C12	1.383 (4)	C21—C22	1.378 (4)
C7—C8	1.386 (4)	C21—H21	0.9300
C7—N5	1.411 (4)	C22—C23	1.378 (4)
C8—C9	1.374 (5)	C22—H22	0.9300
C8—H8	0.9300	C23—C24	1.383 (4)
C9—C10	1.361 (5)	C23—CL2	1.736 (3)
C9—H9	0.9300	C24—H24	0.9300
C10—C11	1.366 (5)	N1—H1A	0.8600
C10—H10	0.9300	N1—H1B	0.8600
C11—C12	1.387 (4)	N2—H2A	0.8600
C11—CL1	1.736 (3)	N2—H2B	0.8600
C12—H12	0.9300	N5—H5	0.8600
C13—N9	1.346 (4)	N6—H6A	0.8600
C13—N6	1.352 (4)	N6—H6B	0.8600
C13—C14	1.378 (4)	N7—H7A	0.8600
C14—C15	1.398 (4)	N7—H7B	0.8600
C14—H14	0.9300	N10—H10A	0.8600
			
N1—C1—N3	116.0 (3)	C18—C17—S2	115.1 (2)
N1—C1—C2	122.9 (3)	C18—C17—H17A	108.5
N3—C1—C2	121.1 (3)	S2—C17—H17A	108.5
C3—C2—C1	118.1 (3)	C18—C17—H17B	108.5
C3—C2—H2	121.0	S2—C17—H17B	108.5
C1—C2—H2	121.0	H17A—C17—H17B	107.5
N2—C3—N4	115.0 (3)	O2—C18—N10	124.6 (3)
N2—C3—C2	122.9 (3)	O2—C18—C17	120.6 (3)
N4—C3—C2	122.0 (3)	N10—C18—C17	114.9 (2)
N3—C4—N4	128.8 (3)	C24—C19—C20	119.2 (3)
N3—C4—S1	111.4 (2)	C24—C19—N10	122.4 (3)
N4—C4—S1	119.8 (2)	C20—C19—N10	118.3 (3)
C6—C5—S1	112.9 (2)	C21—C20—C19	120.6 (3)
C6—C5—H5A	109.0	C21—C20—H20	119.7
S1—C5—H5A	109.0	C19—C20—H20	119.7
C6—C5—H5B	109.0	C22—C21—C20	121.0 (3)
S1—C5—H5B	109.0	C22—C21—H21	119.5
H5A—C5—H5B	107.8	C20—C21—H21	119.5
O1—C6—N5	124.5 (3)	C23—C22—C21	117.8 (3)
O1—C6—C5	120.8 (3)	C23—C22—H22	121.1
N5—C6—C5	114.8 (3)	C21—C22—H22	121.1
C12—C7—C8	120.1 (3)	C22—C23—C24	122.6 (3)
C12—C7—N5	122.3 (3)	C22—C23—CL2	119.1 (3)
C8—C7—N5	117.6 (3)	C24—C23—CL2	118.2 (2)
C9—C8—C7	120.3 (3)	C23—C24—C19	118.8 (3)
C9—C8—H8	119.8	C23—C24—H24	120.6
C7—C8—H8	119.8	C19—C24—H24	120.6
C10—C9—C8	120.3 (4)	C1—N1—H1A	120.0
C10—C9—H9	119.8	C1—N1—H1B	120.0
C8—C9—H9	119.8	H1A—N1—H1B	120.0
C9—C10—C11	119.2 (3)	C3—N2—H2A	120.0
C9—C10—H10	120.4	C3—N2—H2B	120.0
C11—C10—H10	120.4	H2A—N2—H2B	120.0
C10—C11—C12	122.5 (3)	C4—N3—C1	115.3 (2)
C10—C11—CL1	119.5 (3)	C4—N4—C3	114.6 (2)
C12—C11—CL1	118.0 (3)	C6—N5—C7	128.7 (3)
C7—C12—C11	117.5 (3)	C6—N5—H5	115.6
C7—C12—H12	121.2	C7—N5—H5	115.6
C11—C12—H12	121.2	C13—N6—H6A	120.0
N9—C13—N6	115.3 (3)	C13—N6—H6B	120.0
N9—C13—C14	121.8 (3)	H6A—N6—H6B	120.0
N6—C13—C14	122.8 (3)	C15—N7—H7A	120.0
C13—C14—C15	117.4 (3)	C15—N7—H7B	120.0
C13—C14—H14	121.3	H7A—N7—H7B	120.0
C15—C14—H14	121.3	C16—N8—C15	115.7 (3)
N7—C15—N8	116.7 (3)	C16—N9—C13	115.5 (2)
N7—C15—C14	122.0 (3)	C18—N10—C19	129.5 (3)
N8—C15—C14	121.3 (3)	C18—N10—H10A	115.3
N8—C16—N9	128.2 (3)	C19—N10—H10A	115.3
N8—C16—S2	112.7 (2)	C4—S1—C5	103.66 (14)
N9—C16—S2	119.0 (2)	C16—S2—C17	102.99 (14)
			
N1—C1—C2—C3	178.0 (3)	N10—C19—C24—C23	178.0 (3)
N3—C1—C2—C3	−0.6 (4)	N4—C4—N3—C1	2.2 (5)
C1—C2—C3—N2	179.3 (3)	S1—C4—N3—C1	−175.7 (2)
C1—C2—C3—N4	3.1 (4)	N1—C1—N3—C4	179.5 (3)
S1—C5—C6—O1	−93.7 (3)	C2—C1—N3—C4	−1.9 (4)
S1—C5—C6—N5	85.2 (3)	N3—C4—N4—C3	0.1 (4)
C12—C7—C8—C9	2.3 (5)	S1—C4—N4—C3	177.8 (2)
N5—C7—C8—C9	−178.8 (3)	N2—C3—N4—C4	−179.4 (3)
C7—C8—C9—C10	−1.9 (6)	C2—C3—N4—C4	−2.9 (4)
C8—C9—C10—C11	0.6 (6)	O1—C6—N5—C7	2.0 (5)
C9—C10—C11—C12	0.3 (6)	C5—C6—N5—C7	−176.9 (3)
C9—C10—C11—CL1	−179.8 (3)	C12—C7—N5—C6	−20.0 (5)
C8—C7—C12—C11	−1.4 (5)	C8—C7—N5—C6	161.1 (3)
N5—C7—C12—C11	179.7 (3)	N9—C16—N8—C15	2.3 (5)
C10—C11—C12—C7	0.1 (5)	S2—C16—N8—C15	−174.2 (2)
CL1—C11—C12—C7	−179.8 (2)	N7—C15—N8—C16	177.1 (3)
N9—C13—C14—C15	1.8 (5)	C14—C15—N8—C16	−2.7 (5)
N6—C13—C14—C15	179.0 (3)	N8—C16—N9—C13	0.1 (5)
C13—C14—C15—N7	−178.9 (3)	S2—C16—N9—C13	176.4 (2)
C13—C14—C15—N8	0.8 (5)	N6—C13—N9—C16	−179.7 (3)
S2—C17—C18—O2	−112.5 (3)	C14—C13—N9—C16	−2.2 (5)
S2—C17—C18—N10	68.4 (3)	O2—C18—N10—C19	4.6 (5)
C24—C19—C20—C21	−0.7 (4)	C17—C18—N10—C19	−176.3 (3)
N10—C19—C20—C21	−178.3 (3)	C24—C19—N10—C18	1.4 (5)
C19—C20—C21—C22	0.5 (5)	C20—C19—N10—C18	178.8 (3)
C20—C21—C22—C23	−0.1 (5)	N3—C4—S1—C5	−172.4 (2)
C21—C22—C23—C24	−0.1 (5)	N4—C4—S1—C5	9.5 (3)
C21—C22—C23—CL2	177.2 (2)	C6—C5—S1—C4	−88.7 (2)
C22—C23—C24—C19	−0.1 (4)	N8—C16—S2—C17	−172.9 (2)
CL2—C23—C24—C19	−177.5 (2)	N9—C16—S2—C17	10.3 (3)
C20—C19—C24—C23	0.6 (4)	C18—C17—S2—C16	−86.9 (2)

### (II) N-(3-Chlorophenyl)-2-[(4,6-diaminopyrimidin-2-yl)sulfanyl]acetamide Hydrogen-bond geometry (Å, º)

**Table:**

*D*—H···*A*	*D*—H	H···*A*	*D*···*A*	*D*—H···*A*
N5—H5···N4	0.86	2.25	2.962 (3)	140
N10—H10*A*···N9	0.86	2.02	2.826 (3)	157
N1—H1*B*···O1^i^	0.86	2.19	2.931 (4)	145
N2—H2*B*···Cl1^i^	0.86	2.76	3.405 (3)	133
N6—H6*A*···O2^ii^	0.86	2.51	3.340 (4)	162
N6—H6*B*···Cl2^iii^	0.86	2.70	3.556 (3)	176
N7—H7*B*···O2^iii^	0.86	2.24	3.002 (4)	148
N1—H1*A*···N8^iv^	0.86	2.21	3.070 (4)	174
N7—H7*A*···N3^v^	0.86	2.19	3.046 (4)	178

Symmetry codes: (i) *x*, −*y*+3/2, *z*+1/2; (ii) −*x*+2, −*y*+1, −*z*; (iii) *x*, −*y*+3/2, *z*−1/2; (iv) *x*−1, −*y*+3/2, *z*+1/2; (v) *x*+1, −*y*+3/2, *z*−1/2.
